# Prenatal MRI abdominal volumetrics and outcomes after early surgery or paint-and-wait for giant omphalocele

**DOI:** 10.3389/fped.2026.1912189

**Published:** 2026-08-03

**Authors:** Daniel R. Liesman, Steven T. Papastefan, Katherine C. Ott, Elissa Abou Khalil, Saad Ranginwala, William Marriott, Meghan A. Coghlan, Amir M. Alhajjat, Aimen F. Shaaban

**Affiliations:** 1 Chicago Institute for Fetal Health, Ann and Robert H. Lurie Children’s Hospital, Chicago, IL, United States; 2 Department of Surgery, Northwestern University Feinberg School of Medicine, Chicago, IL, United States; 3 Department of Pediatrics, Northwestern University Feinberg School of Medicine, Chicago, IL, United States

**Keywords:** anomaly, giant omphalocele, MRI, outcomes, paint-and-wait, primary closure

## Abstract

Giant Omphalocele (GO) poses significant challenges in neonatal management. Standard treatment is paint-and-wait with delayed fascial closure at 1–2 years-of-age. Alternatively, early surgery entails neonatal sac removal with either primary fascial closure or staged GORE-TEX patch-assisted closure. We compared outcomes between early surgery and paint-and-wait and evaluated whether prenatal MRI volumetrics can identify candidates for primary closure. We performed a retrospective study (2007–2024) of newborns with GO (sac diameter ≥5 cm with liver). For early surgery patients, prenatal MRI volumetric analysis measured intra-abdominal (IAV) and eviscerated organ volumes (EOV) to generate a novel evisceration index (Evisceration Index = (EOV/(EOV + IAV)) × 100. Forty-three infants met inclusion (25 early, 18 paint-and-wait). Groups were similar for birth <34 weeks, weight, sac diameter, and associated anomalies. No patients required ECMO. Mortality was comparable (8.0% vs. 11.1%; *p* = 1.00). Early surgery achieved earlier fascial closure (27 vs. 464 days; *p* < 0.01) with 34.8% closed at the first operation. Tracheostomy was less common with early surgery (4.0% vs. 33.3%; *p* = 0.02). Among early surgery infants with prenatal MRI (*n* = 21), those achieving primary closure had a lower evisceration index (16.0% vs. 37.0%; *p* < 0.01). ROC analysis identified an evisceration index cut point of 26%, above which staged closure was necessary. Early surgery results in earlier fascial closure with lower tracheostomy rates. Prenatal MRI volumetric analysis demonstrated potential utility for identifying GO patients who may be able to undergo primary fascial closure with a single operation supporting the possibility of a more individualized approach to prenatal counseling.

## Introduction

Omphalocele is a rare congenital defect of the abdominal wall with a broad spectrum of severity ranging from a small defect at the base of the umbilical cord containing a few loops of intestine to a giant omphalocele (GO) that contains varying degrees of liver, stomach, intestines, spleen and even kidney (1). GO is frequently associated with pulmonary hypoplasia, cardiac anomalies, and other comorbidities (2). The management of omphalocele is largely dependent on the size of the defect and contents of the sac. Primary fascial closure early in neonatal life is the preferred approach for smaller omphaloceles whereas GO is typically treated with non-operative wound care due to concern for abdominal-respiratory competition with early surgical closure (3). This “paint-and-wait” approach permits delayed fascial closure once the sac has epithelialized and the infant has grown sufficiently to tolerate reduction of the herniated viscera. Consequently, most pediatric surgeons favor this approach when tasked with managing a GO (4).

Two systematic reviews comparing early surgery with paint-and-wait reported shorter time to full enteral feeds and lower mortality among escharization patients (5, 6). However, these findings were limited by heterogeneity among the early surgery patients, small sample sizes, and the inclusion of older studies that do not adequately capture advancements in neonatal perioperative care. Currently, there is insufficient evidence to support that early surgical management is inferior to the paint-and-wait approach highlighting a knowledge gap in the counseling and care of these patients. More recent reports suggest that early surgery may offer several benefits over a paint-and-wait approach (7, 8). One obvious advantage of early surgery is the elimination of meticulous long-term wound care that is required for escharization and epithelialization of the sac (9). Furthermore, early fascial closure may improve respiratory mechanics in neonates who are obligate abdominal breathers, potentially help avoid the unresolved challenges of cases of severe viscero-abdominal disproportion, and improve health-related quality of life indices in early life (10–13).

Due to disparate strategies for managing GO, we investigated how early surgery with either primary or staged repair influences neonatal outcomes in comparison to management with paint-and-wait. We hypothesized that early surgery would enable earlier fascial closure without increasing complications in the newborn period. Additionally, we examined prenatal MRI data for those patients who underwent early surgery to determine predictors of primary closure after birth.

## Methods

### Study cohort

We performed a retrospective cohort study of infants with GO treated at our institution between 2007 and 2024. The study was determined to be exempt by the Institutional Review Board (IRB 2020-3250). Inclusion criteria were a diagnosis of GO defined as an omphalocele containing liver with a sac diameter ≥ 5 cm (14–16). Neonates who died before initiation of a treatment plan due to severe prematurity or associated anomalies, or whose family's elected withdrawal of care in the early neonatal period were excluded. The final patients were categorized as either early surgery, which included both primary closure and staged repair or paint-and-wait. Management strategy reflected an institutional shift occurring around 2017 with paint-and-wait the predominant approach before this while early surgery became the standard after. As a result, the choice of treatment pathway was determined primarily by the era the patient presented rather than by individual patient selection.

**Figure 1:**
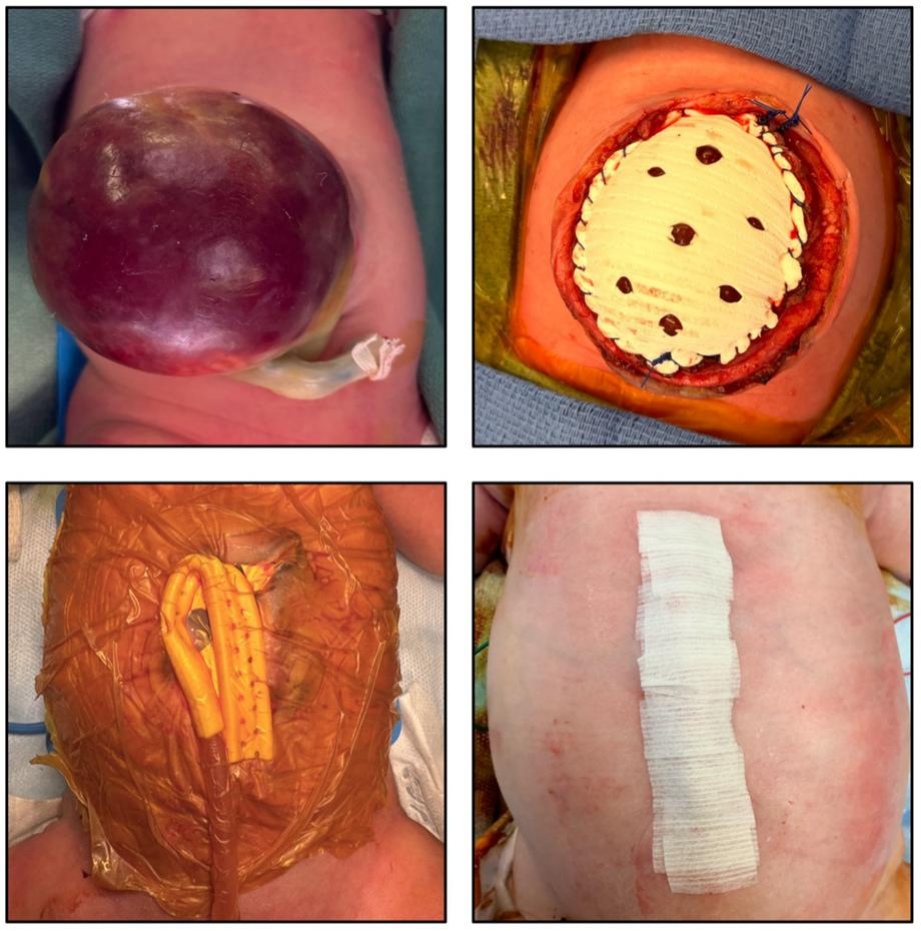
Operative steps for staged closure. A patient with a giant omphalocele (top left) had a GORE-TEX patch sewn to the surrounding fascia (top right) after removal of the amnion. A sterile iodine-impregnated dressing was then applied with suction drainage provided by a Jackson-Pratt drain (bottom left). The dressing was removed for intermittent tightening of the patch allowing for final closure on day-of-life 33.

At our institution, staged repair utilizes a stepwise approach first by removal of the amnion and then with the creation of a Dual-Mesh GORE-TEX (Gore and Associates, Flagstaff, AZ) patch with negative pressure dressing followed by serial bedside surgical reduction. This is achieved by excising an ellipse in the center of the GORE-TEX and then sewing that defect closed to provide traction on the fascial edges (Figure 1) which can be done bedside in the NICU. While the patch is in place, an antimicrobial dressing is used with a Jackson-Pratt drain to provide drainage of the peritoneal fluid that accumulates. Definitive fascial closure is then performed once adequate abdominal domain has been achieved. In some cases, a Bentec silo (Bentec Med, Woodland, CA) is used at the initial operation prior to subsequent creation of the GORE-TEX patch. Primary fascial closure was attempted when feasible. However, if primary closure was not achievable then the patient was shunted down the staged pathway. Lastly, in recent years, botulinum toxin chemo-denervation of the lateral abdominal wall muscles (external oblique, internal oblique and transversus abdominis) has been used as an adjunct to increase abdominal domain by relaxation.

### Definitions

For this study the following patient characteristics and outcomes were defined according to existing literature and expert consensus among the authors:

- Sac size and contents—Measured and described in the operating room at the time of early surgery or based on the physical exam after birth performed by the Neonatology or Pediatric Surgery team. If neither were available then the most recent third trimester prenatal imaging was used. We chose to use sac diameter as the metric for GO inclusion as opposed to fascial defect size for two reasons: (1) Some of our patients had mushroom defects where almost the entirety of the liver and much of the bowel were herniated through a relatively small fascial defect. Supplementary Figure S1 shows an example of an unrepaired mushroom defect; (2) The size of the fascial defect could not be reliably assessed in many patients managed with paint-and-wait because the only consistently available measurement at delivery was sac diameter determined by physical examination.
- Pulmonary hypertension—Defined as the presence of a tricuspid regurgitant jet with an estimated systolic pulmonary artery pressure > two-thirds of the systemic systolic blood pressure, and/or persistent right-to-left or bidirectional flow at the ductal or atrial level beyond 24 h of life, and/or clinical evidence of pre- vs. post- SpO_2_ difference greater than 10% necessitating the use of pulmonary vasodilators (17–20).
- Length of intubation—Number of days spent intubated following initial intubation for respiratory distress. For patients who underwent tracheostomy, the day of tracheostomy placement was considered the end of the intubation period. Patients who were palliatively extubated or those who died while intubated were excluded from this metric.
- Operations during staged closure—Silo placement, patch placement or serial reduction, or fascial closure each counted as one operation.
- Sepsis—Encompassed both bacteremia with positive blood cultures or antibiotic treatment for ≥5 days for an infectious source as determined by the treating team and not for prophylaxis (7). Abdominal sepsis was defined as either positive peritoneal fluid cultures or clinical concerns for infection including purulent or foul smelling discharge from the abdomen or cellulitis surrounding the sac or patch prompting treatment.

### Postnatal characteristics and outcomes

Patient data for both baseline characteristics and outcomes were extracted from the medical record. Characteristics included birth before 34 weeks' gestation, maternal age at delivery, delivery method, sex, and birth weight. Outcomes data included mortality, length of hospital stay, time to fascial closure, requirement of extracorporeal membrane oxygenation (ECMO), omphalocele sac rupture, the development of abdominal compartment syndrome (ACS), feeding outcomes, and hernia formation after repair. Patient weights were converted to gestational age-adjusted Z-scores using World Health Organization standards.

### MRI analysis

For patients in the early surgery cohort who underwent prenatal MRI, volumetric measurements of intra-abdominal volume (IAV) and eviscerated organ volume (EOV) were performed using Visage 7 (Visage Imaging, San Diego, California). All prenatal MRIs were acquired via a standardized institutional protocol with T2-weighted sequences. Volumetric assessment was performed by the fetal research fellow (D.R.L.) with assistance from a trained fetal radiologist (S.R.) Intra-abdominal borders were defined as follows: anterior—abdominal wall; posterior—paraspinal musculature and vertebral column; lateral—lateral abdominal wall musculature; superior—the most caudal axial slice in which both lungs were visible; and inferior—the most cephalad axial slice in which the bladder is visible. Intra-abdominal volumes were segmented and calculated from axial images. Eviscerated organ volume within the GO sac was measured using either axial or sagittal sequences, depending on which provided the most complete visualization of the sac contents. A novel evisceration index was then calculated using the following equation to assess the relative volume of eviscerated viscera to total volume and to explore its association with the feasibility of primary closure vs. the need for staged repair:

Evisceration Index = (*EOV* / (*EOV* + *IAV*)) × 100 (1)

### Statistical analysis

Continuous data is reported as median (IQR). Categorical variables are displayed as frequency and percentage. Normality of continuous variables was assessed using the Shapiro–Wilk test. Continuous variables were compared with the Student's t-test or Mann Whitney U test where appropriate based on normality. Categorical variables were compared with the Fisher's Exact test or Chi Square test where appropriate. Significance was considered to be a *p*-value ≤ 0.05. The optimal threshold was defined as the threshold yielding the maximum mean Youden's J across candidate thresholds. This criterion prioritizes the identification of staged vs. primary closure patients by maximizing combined sensitivity and specificity. The optimal-threshold output included the selected threshold and Youden's J, AUC, sensitivity, and specificity. All statistical analysis was performed in GraphPad Prism (GraphPad, Boston, MA).

## Results

### Cohort identification and baseline characteristics

**Figure 2:**
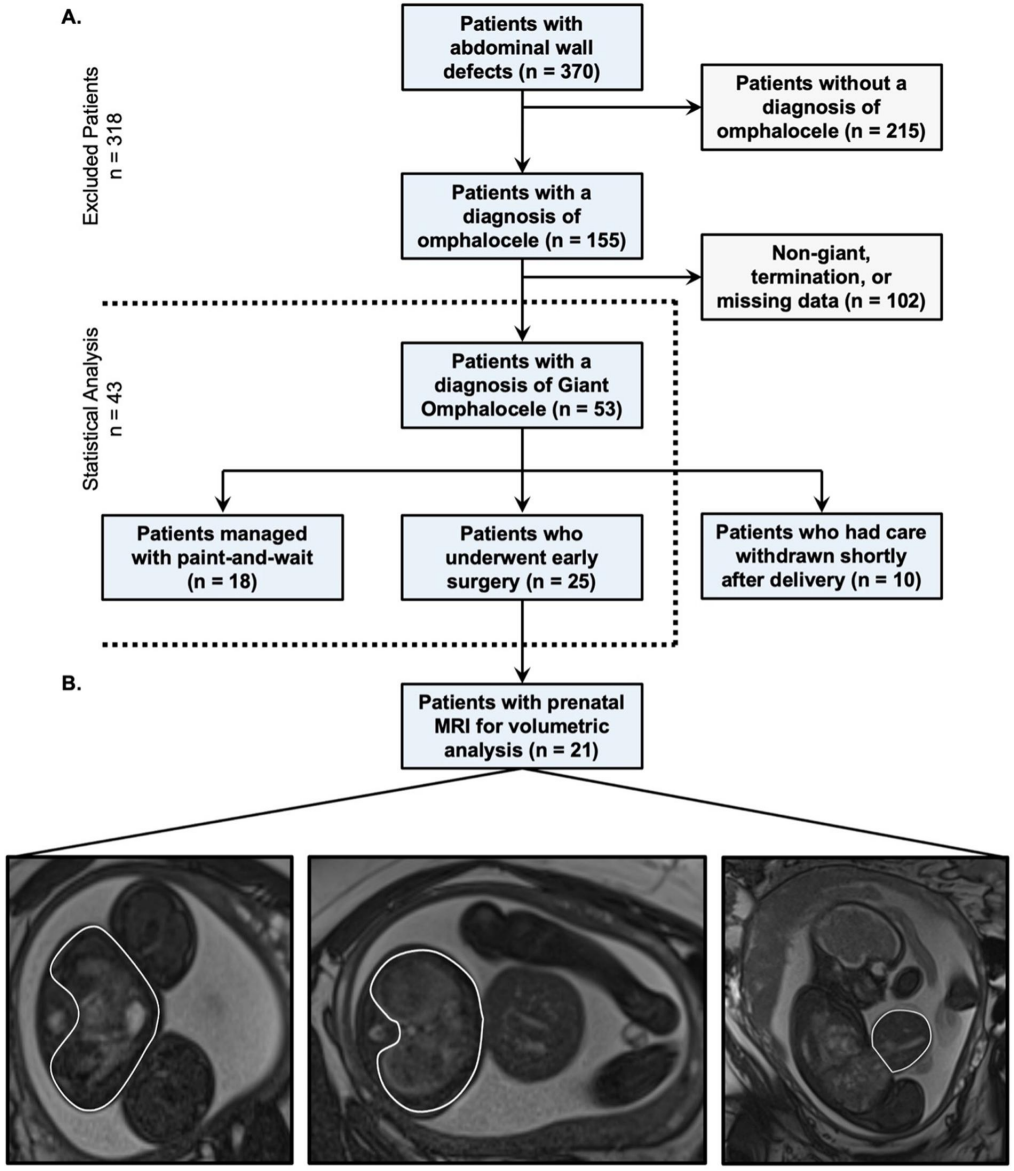
Patient selection flowchart with exclusions. **(A)** Flowchart showing the final patient cohort. **(B)** Three MRI images illustrating the technique for fetal MRI volumetry in GO with the first image showing the most superior visible portion of the bladder and the second image showing the superior portion of the abdomen with the inferior bases of both lungs visible. The third image shows the outline of the sac measurement. The first two images represent the intra-abdominal volume (IAV) and the third image represents the eviscerated organ volume (EOV).

A total of 370 patients were identified with abdominal wall defects in the study period (Figure 2A). Of these, 155 (41.9%) had an omphalocele and 53 (14.3%) met criteria for GO. Of the neonates with GO, ten (18.9%) were excluded due to withdrawal of care after delivery and prior to initiation of therapy. The remaining 43 neonates met criteria for analysis with 25 (58.1%) managed with early surgery and 18 (41.9%) managed with paint-and-wait. The early surgery patients were selected for the MRI analysis with 21 (84.0%) having undergone a prenatal MRI and were therefore included in the volumetric analysis (Figure 2B).

Baseline maternal and perinatal characteristics were similar between groups (Table 1). There was no difference in gestational age at delivery before 34 weeks (4.0% of early surgery and 11.1% of paint-and-wait patients; *p* = 0.56), and all infants were delivered by cesarean section. Birthweight trended higher in the early surgery cohort [3.0 [2.8, 3.4] vs. 2.8 [2.3, 3.3] kg; *p* = 0.08]. Sac diameter also showed a non-significant trend toward larger defects in the early surgery group [8.0 [6.4, 9.6] vs. 7.0 [5.8, 7.9] cm; *p* = 0.08]. There was no difference in sac contents between the two groups. Severe cardiac anomalies were present only in the early surgery group although this was not significantly different from the paint-and-wait group (16.0% vs. 0%; *p* = 0.13) (Table 1). Non-severe cardiac defects, genetic anomalies, and pulmonary hypertension occurred at similar frequencies (all *p* > 0.05).

**Table 1:** Prenatal, delivery, and postnatal patient baseline characteristics.

Baseline characteristics	Paint-and-wait	Early surgery	*p*-value
	*N* = 18	*N* = 25	
Maternal Age at Birth (Years)	32.6 (26.9, 37.7)	34.0 (30.5, 36.8)	0.49
Polyhydramnios^a^	3 (18.8%)	3 (12.5%)	0.67
Born Before 34 Weeks	2 (11.1%)	1 (4.0%)	0.56
Birth Weight (kg)	2.8 (2.3, 3.3)	3.0 (2.8, 3.4)	0.08
Sex (Female)	8 (44.4%)	13 (52.0%)	0.62
Delivery Method (C-Section)	18 (100%)	25 (100%)	1.00
Sac Diameter (cm)^b^	7.0 (5.8, 7.9)	8.0 (6.4, 9.6)	0.08
Sac Contains Stomach	7 (38.9%)	11 (44.0%)	0.74
Sac Contains Bowel	10 (55.6%)	15 (60.0%)	0.77
Sac Contains Gallbladder	2 (11.1%)	7 (28.0%)	0.26
Pulmonary Hypertension	7 (38.9%)	6 (24.0%)	0.29
Severe Congenital Heart Anomaly	0 (0%)	4 (16.0%)	0.13
Doublet Outlet Right Ventricle	0 (0%)	1 (4.0%)	1.00
Tetralogy of Fallot	0 (0%)	1 (4.0%)	1.00
Coarctation of the Aorta	0 (0%)	1 (4.0%)	1.00
Septal Defect Requiring Closure	0 (0%)	1 (4.0%)	1.00
Non-Severe Congenital Heart Anomaly	11 (61.1%)	16 (64.0%)	0.85
Atrial Septal Defect	5 (27.8%)	2 (8.0%)	0.11
Ventricular Septal Defect	3 (16.7%)	2 (8.0%)	0.63
Patent Ductus Arteriosus	8 (44.4%)	15 (60.0%)	0.31
Dextrocardia	1 (5.6%)	0 (0%)	0.42
Genetic Anomaly	2 (11.1%)	6 (24.0%)	0.43
Non-Cardiac Congenital Defects	7 (38.9%)	8 (32.0%)	0.64

a Data available for 15 paint-and-wait and 24 early surgery patients.

b Data available for 17 paint-and-wait patients.

### Patient outcomes

During the index hospital stay*,* mortality occurred in 2 patients (8.0%) in the early surgery group and 2 patients (11.1%) in the paint-and-wait group (*p* = 1.00). The two early surgery deaths included one patient with severe fetal growth restriction and pulmonary hypertension who died from undifferentiated shock with a GORE-TEX patch in place, and one patient with Tetralogy of Fallot who was transitioned to wound vacuum management during staged closure from a necrotizing lung infection. In the paint-and-wait group, one patient died from septic shock complicated by renal failure and severe electrolyte abnormalities and one patient with severe pulmonary hypertension died after the family withdrew care. No neonates in either cohort required ECMO. APGARs, sac rupture, and delivery room intubation for respiratory distress were similar between the groups. The early surgery group had lower tracheostomy rates than the paint-and-wait group (4.0% vs. 33.3%; *p* = 0.02). Overall sepsis rates occurred in 48.0% of early surgery patients and 66.7% of paint-and-wait patients and (*p* = 0.22). However, bacteremia occurred less frequently in early surgery patients (0% vs. 27.8%; *p* = 0.01). Oxygen requirement at 30 days-of-life, length of intubation, and rates of tracheobronchomalacia were similar between both groups (Table 2). Time to first feed [9 [5, 33] days vs. 3 [1,6] days; *p* < 0.01] and time to full feeds [51 [18, 61] vs. 16 [8, 31] days; *p* = 0.02] both occurred later in the early surgery group with higher TPN utilization (96.0% vs. 66.7%; *p* = 0.02). However, Z-scores for weight at 3 and 6 months were no different.

**Table 2:** Outcomes between paint-and-wait and early surgery groups.

Outcomes	Paint-and-wait	Early surgery	*P*-value
	*N* = 18	*N* = 25	
1 min APGAR^a^	6 (3, 8)	8 (6, 8)	0.46
5 min APGAR^a^	8 (6, 9)	8 (7, 9)	0.18
Mortality	2 (11.1%)	2 (8.0%)	1
Need for ECMO	0 (0%)	0 (0%)	1
Sac rupture	3 (16.7%)	5 (20.0%)	1
Delivery room intubation for distress	8 (44.4%)	9 (36.0%)	0.58
Length of intubation (days)^b^	58 (9, 123)	43 (10, 73)	0.93
O_2_ requirement at day 30 for survivors	9 (52.9%)	16 (66.7%)	0.37
Need for tracheostomy	6 (33.3%)	1 (4.0%)	0.02
Sepsis during hospital stay	12 (66.7%)	12 (48.0%)	0.22
Abdominal	2 (11.1%)	7 (28.0%)	0.26
Genitourinary	4 (22.2%)	1 (4.0%)	0.14
Bacteremia	5 (27.8%)	0 (0%)	0.01
Pulmonary	5 (27.8%)	6 (24.0%)	0.78
Unknown	4 (22.2%)	2 (8.0%)	0.22
Length of stay for survivors (days)	43 (29, 294)	79 (45, 165)	0.88
Days to definitive closure^c^	464 (326, 910)	27 (1, 67)	<0.01
Number of operations for closure^c^			0.02
1	12 (80.0%)	8 (34.8%)	–
2	1 (6.7%)	3 (13.0%)	–
≥3	2 (13.3%)	12 (52.2%)	–
Developed ACS	0 (0%)	2 (8.0%)	0.5
Tracheobronchomalacia	2 (11.1%)	4 (16.0%)	1
Required pulmonary vasodilators	7 (38.9%)	3 (12.0%)	0.07
Need for TPN	12 (66.7%)	24 (96.0%)	0.02
Days to first feed^d^	3 (1, 6)	9 (5, 33)	<0.01
Days to full feeds^e^	16 (8, 31)	51 (18, 61)	0.02
Peak bilirubin during index stay	7.8 (3.8, 11.1)	7.4 (5.2, 9.9)	0.89
Weight Z-Score 3 months^f^	−0.93 (−1.90, −0.37)	−1.12 (−1.61, −0.32)	0.57
Weight Z-Score 6 months^g^	−1.05 (−1.71, 0.84)	−1.23 (−1.83, −0.36)	0.63
Hernia formation after repair^e^	2 (13.3%)	3 (13.0%)	1

a Data available for 17 paint-and-wait patients.

b Patients with in-hospital mortality were excluded. The date of tracheostomy was the date of extubation.

c Data available for 15 paint-and-wait patients.

d Data available for 16 paint-and-wait and 24 early surgery patients.

e Data available for 15 paint-and-wait and 23 early surgery patients.

f Data available for 14 paint-and-wait and 23 early surgery patients.

g Data available for 16 paint-and-wait and 21 early surgery patients.

Among survivors, there was no statistically significant difference in length of stay [79 [45, 165] vs. 43 [29, 294] days; *p* = 0.88]. As expected, closure-related outcomes differed significantly. Of the early surgery patients who achieved closure, eight (34.8%) were closed in a single operation while 15 (65.2%) required staged closure (Table 2). Early surgery patients achieved fascial closure significantly earlier than paint-and-wait patients [27 [1, 67] vs. 464 [326, 910] days; *p* < 0.01]. Two of the 25 (8.0%) early surgery patients developed abdominal compartment syndrome requiring patch loosening (both were staged patients). Both cases were identified with early postnatal monitoring that responded immediately to patch loosening without sequelae.

### Prenatal MRI volumetric analysis

**Figure 3:**
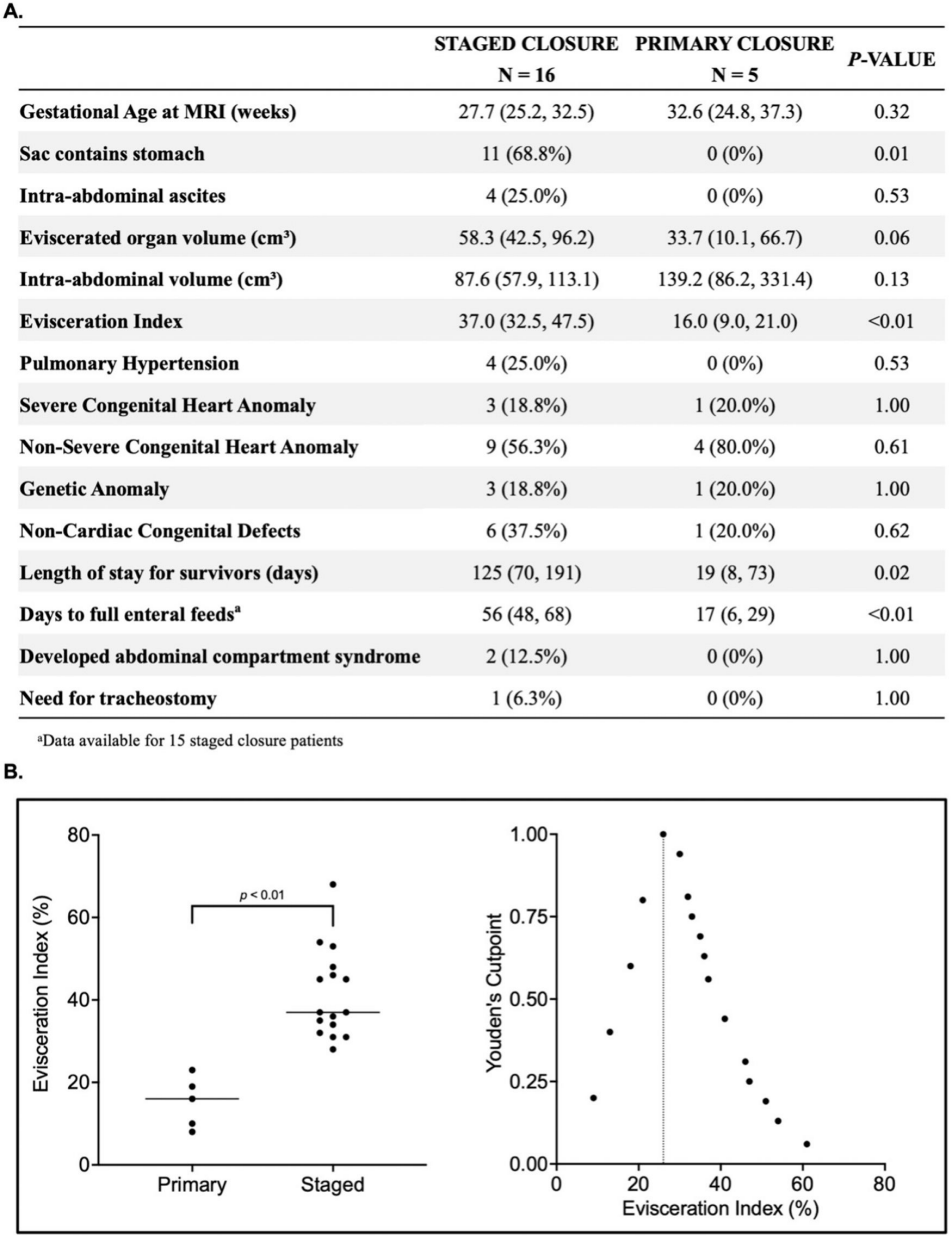
Volumetric prenatal MRI analysis reveals stark differences between primary and staged closure patients. **(A)** Baseline characteristics and outcomes of the 21 early surgery patients with prenatal MRIs available. **(B)** Scatter plot showing a difference in the evisceration index between patients closed primarily and those who required staged closure and a graph depicting 26% as the threshold to discriminate primary and staged closure.

There were 21 neonates in the early surgery group (16 staged closure and 5 primary closure) with prenatal MRIs. There were no differences in the gestational age at which the MRIs were done (Figure 3A). However, the evisceration index was markedly lower in primary closure neonates in comparison to those who required staged closure (16.0% vs. 37.0%; *p* < 0.01). Additionally, no neonate who underwent primary closure had their stomach in the sac (0% vs. 68.8%; *p* = 0.01). Youden's optimal cut point (Youden's J = 1.00) identified an evisceration index cutoff of ≥26% for those patients who required staged closure (Figure 3B).

## Discussion

This study compared outcomes for patients with GO managed with either paint-and-wait or with an early surgery approach. We found that infants in the early surgery group achieved definitive fascial closure substantially earlier, without an associated increase in major complications. Notably, hospital length-of-stay, while influenced by clinical, nutritional, and social factors, was similar between groups suggesting that early surgery might not add significant morbidity (21). The paint-and-wait cohort, however, demonstrated a significantly higher rate of tracheostomy placement and more episodes of bacteremia. Importantly, 34.8% of the early surgery patients could be primarily closed at the first operation even with significant liver herniation. The prenatal MRI volumetric analysis suggests that primary closure can be predicted before birth. Together, these data support that early surgical management may be able to be performed safely in selected neonates with GO.

Several prenatal imaging parameters have been utilized to predict neonatal outcomes or the likelihood of primary closure for patients with an omphalocele. Most studies in this area have relied on ultrasound-based measures including the diameter and/or circumference of the omphalocele in relation to the abdomen (22–24). Fetal MRI, by contrast, has shown prognostic value in GO, particularly through assessment of observed-to-expected total lung volume and has become a routine component of prenatal evaluation (25, 26). Our findings extend the prognostic utility of this test by demonstrating that abdominal and sac volumetric analysis can specifically predict the most likely type of postnatal surgical repair. However, the MRI analysis, particularly on those who underwent early primary closure, was based on a small number of patients and must be considered exploratory. Additionally, MRI data was only available for meaningful calculation from the early surgery group. External validation in a larger, prospective cohort is necessary to better delineate cutoffs and optimal gestational age for calculation before this threshold can be used clinically to inform care.

A recent publication examining prenatal MRI predictors for neonatal morbidity and mortality identified the presence of the stomach in the sac as a marker for both a need for paint-and-wait and increased mortality (27). However, the current data reveals similar rates of stomach herniation for paint-and-wait and early surgery cohorts without an increase in mortality. Additionally, stomach herniation seemed to preclude the possibility of primary closure at the first operation necessitating staged surgical reduction. This suggests that pursuing early surgery when the stomach is herniated is feasible but primary closure will be unlikely. Future studies of partial or complete stomach herniation in giant omphalocele will likely delineate the prognostic utility of this straightforward anatomic observation. Collectively, these findings inform prenatal counseling about the anticipated postnatal surgical management. With favorable prenatal MRI parameters (e.g., evisceration index less than 26% and stomach in the abdomen), surgeons may reasonably counsel about the likelihood for early sac excision and primary fascial closure at the first neonatal operation.

There has been growing momentum toward earlier definitive repair, facilitated by new compressive and closure techniques. A recent registry from the Midwest Pediatric Surgery Consortium reported outcomes with the use of DuoDERM (Convatec, London, United Kingdom) silo, paint-and-wait, operative silo, and alternative techniques in the management of GO (4). Here, silo-assisted compression (DuoDERM or operative silo) was performed in a minority of patients (23/117) with higher overall rates of fascial closure (83% and 91% respectively) than with paint-and-wait or the use of alternative compression techniques (69% and 64%, respectively). However, none of the patients included in the registry underwent primary closure. A direct comparison with the current report is difficult given the absence of a consistent definition of a GO. Establishing standardized diagnostic criteria would enhance the comparability of studies and help determine best practices for integrating imaging with postnatal surgical planning.

One of the most striking findings in our results was the differing patterns of infectious outcomes between the two groups. Infection remains a major cause of morbidity and mortality in GO, and these infants are uniquely vulnerable to bacteremia (3, 28, 29). Although there were no differences in overall sepsis rates during the index hospitalization, bacteremia episodes occurred significantly more often in the paint-and-wait group. This may reflect the prolonged exposure of the sac to the external environment with likely colonization of the eschar and the potential for systemic inflammation that may trigger indolent pulmonary hypertension as previously postulated (30). In contrast, the reduction and coverage of the viscera with early surgery in the current report with the use of a negative pressure GORE-TEX patch and antibiotic prophylaxis may confer protection from bacterial translocation, colonization and chronic systemic inflammation that offset the infectious risks from increased TPN utilization and prolongation of central venous access (6, 31). Ultimately, these parameters need further investigation in a larger multi-institutional study to understand the true infectious burden with either technique.

The stark differences in tracheostomy rates between the groups warrants careful interpretation. While we hypothesize that early fascial closure may improve respiratory mechanics, causality cannot be established from this retrospective data. The era-based separation and smaller sample size introduce confounders that limit any conclusion mechanism. Nevertheless, prior studies lend credence to this hypothesis. Nakayama et al. examined pulmonary dysfunction in neonates before and after closure of abdominal wall defects including “large” omphaloceles and demonstrated that forced vital capacity was 50% of healthy infants when standardized to body weight in the first 2 days after closure (32). Importantly, within 4 weeks of closure, forced vital capacity improved to levels comparable to healthy infants suggesting a favorable impact on respiratory recovery. Additionally, Dimitriou et al. reported that infants after omphalocele repair generated normal maximum inspiratory pressure and transdiaphragmatic pressures when compared with age-matched controls (33). Taken together, the information from these studies suggest that the early surgery patients in the current study may have experienced long term respiratory benefit although this is speculative and the mechanism underlying these differences will need to be better delineated.

The risks of recurrent anesthetic exposure or prolonged sedation is an additional consideration for patients undergoing early surgery for GO. Although 34.8% of early surgery patients were able to undergo primary closure at the initial operation, 65.2% required some form of staged closure exposing them to recurrent anesthetic or sedation procedures. While current data are inconclusive, concerns remain that repetitive anesthetic exposure during childhood may pose risks for long term neurocognitive development (34). However, this possibility must be balanced against the meaningful benefits of early abdominal wall closure.

There are several limitations of this study that may affect the interpretation of the results. First, although the current report is among the largest reports of prenatally diagnosed GO, the overall study size remains small. Further, the absence of a consensus definition of GO limits the comparability of our results to other reported series. The patients in this report were also cared for over a span of 17 years where numerous advances in prenatal diagnosis and newborn care for GO have simultaneously evolved. These advances may have differentially benefited the early surgery cohort and cannot be fully controlled for given the retrospective nature of this study. Although treatment group was largely related to the era in which the patient presented, residual confounding from baseline differences cannot be excluded. For example, the early surgery group trended towards larger sac diameters and included all patients with severe congenital cardiac anomalies. Although these were not statistically significant, they may represent meaningful heterogeneity between groups. Additionally, the evisceration index was calculated only for patients in the early surgery group, as prenatal MRI was not routinely performed during the earlier era when paint-and-wait was the predominant strategy (only two paint-and-wait patients had MRIs available for review). Future prospective studies may enable a more complete evaluation of this index as a clinical tool. Lastly, there were only five patients who underwent primary closure which limits the applicability of the ROC-derived threshold of 26%. The perfect discrimination observed is likely a statistic artifact from the small sample size and should not be interpreted as evidence of a perfect discriminator. This result should therefore be considered exploratory until further validation. Ultimately, many of these limitations will only be addressed with a contemporaneous prospective multi-center study.

## Conclusion

In summary, the findings from this single-center review suggest that early surgery has similar outcomes to a paint-and-wait approach with the benefit of early fascial closure, lower tracheostomy rates and fewer episodes of bacteremia despite a longer need for TPN supplementation. In particular, there may be a subset of patients who would traditionally have been managed with paint-and-wait that could be closed in a single operation and we suggest that a prenatal evisceration index may potentially be useful in this regard. Ultimately, neonatal management of GO may improve with the incorporation of early surgical closure as a potential treatment option.

## Data Availability

The raw data supporting the conclusions of this article will be made available by the authors, upon reasonable request.

## References

[1] Verla MA Style CC Olutoye OO. Prenatal diagnosis and management of omphalocele. Semin Pediatr Surg. (2019) 28(2):84–8. 10.1053/j.sempedsurg.2019.04.007 31072463

[2] Fogelström A Caldeman C Oddsberg J Löf Granström A Mesas Burgos C. Omphalocele: national current birth prevalence and survival. Pediatr Surg Int. (2021) 37(11):1515–20. 10.1007/s00383-021-04978-z 34392395 PMC8520864

[3] Wagner JP Cusick RA. Paint and wait management of giant omphaloceles. Semin Pediatr Surg. (2019) 28(2):95–100. 10.1053/j.sempedsurg.2019.04.005 31072465

[4] Stetson A Leonard S Flynn-O’Brien K Goldstein S Wright T Downard C. A multi-institutional comparison of management techniques for infants with giant omphalocele. J Pediatr Surg. (2025):162822. 10.1016/j.jpedsurg.2025.162822 41260508

[5] Bauman B Stephens D Gershone H Bongiorno C Osterholm E Acton R. Management of giant omphaloceles: a systematic review of methods of staged surgical vs. nonoperative delayed closure. J Pediatr Surg. (2016) 51(10):1725–30. 10.1016/j.jpedsurg.2016.07.006 27570242

[6] Pan R Zhou Z Li Z Cen J Pang F Luo Y. Clinical application and systematic review of waiting treatment for giant omphaloceles. BMC Pediatr. (2025) 25(1):806. 10.1186/s12887-025-06206-2 41073936 PMC12514786

[7] Nolan HR Wagner ML Jenkins T Lim F-Y. Outcomes in the giant omphalocele population: a single center comprehensive experience. J Pediatr Surg. (2020) 55(9):1866–71. 10.1016/j.jpedsurg.2020.04.019 32475506

[8] Roux N Jakubowicz D Salomon L Grangé G Giuseppi A Rousseau V. Early surgical management for giant omphalocele: results and prognostic factors. J Pediatr Surg. (2018) 53(10):1908–13. 10.1016/j.jpedsurg.2018.04.036 29803304

[9] Dorterler ME. Management of giant omphalocele leading to early fascial closure. Cureus. (2019) 11(10):e5932. 10.7759/cureus.5932 31788389 PMC6858265

[10] Fang E Bailey KA Choi M. Repair of a giant omphalocele defect using tissue expansion and Gore-Tex mesh following loss of durable repair with Alloderm: a case report. J Pediatr Surg Case Rep. (2024) 101:102768. 10.1016/j.epsc.2023.102768

[11] Adetayo OA Aka AA Ray AO. The use of intra-abdominal tissue expansion for the management of giant omphaloceles: review of literature and a case report. Ann Plast Surg. (2012) 69(1):104–8. 10.1097/SAP.0b013e31822128f5 21659845

[12] Wiegandt FC Biegger D Fast JF Matusiak G Mazela J Ortmaier T. Detection of breathing movements of preterm neonates by recording their abdominal movements with a time-of-flight camera. Pharmaceutics. (2021) 13(5):721. 10.3390/pharmaceutics13050721 34068978 PMC8156597

[13] Wang P Jin H Guan L Gao M Shao Y Faizan A. Proxy-reported health-related quality of life in children with omphalocele: a cross-sectional study in China. Transl Pediatr. (2026) 15(1):3. 10.21037/tp-2025-aw-678 41657450 PMC12877923

[14] Whitehouse JS Gourlay DM Masonbrink AR Aiken JJ Calkins CM Sato TT. Conservative management of giant omphalocele with topical povidone-iodine and its effect on thyroid function. J Pediatr Surg. (2010) 45(6):1192–7. 10.1016/j.jpedsurg.2010.02.091 20620319

[15] Wakhlu A Wakhlu AK. The management of exomphalos. J Pediatr Surg. (2000) 35(1):73–6. 10.1016/S0022-3468(00)80017-2 10646778

[16] Pandey V Gangopadhyay AN Gupta DK Sharma SP Kumar V. Non-operative management of giant omphalocele with topical povidone-iodine and powdered antibiotic combination: early experience from a tertiary centre. Pediatr Surg Int. (2014) 30(4):407–11. 10.1007/s00383-014-3479-9 24509569

[17] Liu T-X Du L-Z Ma X-L Chen Z Shi L-P. Giant omphalocele associated pulmonary hypertension: a retrospective study. Front Pediatr. (2022) 10:940289. 10.3389/fped.2022.940289 36160768 PMC9505988

[18] Varghese NP Austin ED Galambos C Mullen MP Yung D Guillerman RP. An interdisciplinary consensus approach to pulmonary hypertension in developmental lung disease. Eur Respir J. (2024) 64(3):2400639. 10.1183/13993003.00639-2024 39147412 PMC11424926

[19] Dal Col AK Bhombal S Tacy TA Hintz SR Feinstein J Altit G. Comprehensive echocardiographic assessment of ventricular function and pulmonary pressure in the neonatal omphalocele population. Am J Perinatol. (2021) 38(1):e109–e115. 10.1055/s-0040-1708048 32198744

[20] Shiraishi H Yanagisawa M. Bidirectional flow through the ductus arteriosus in normal newborns: evaluation by Doppler color flow imaging. Pediatr Cardiol. (1991) 12(4):201–5. 10.1007/BF02310566 1946007

[21] Healy GL Stuart CM Dyas AR Bronsert MR Meguid RA Anioke T. Association between postoperative complications and hospital length of stay: a large-scale observational study of 4,495,582 patients in the American college of surgeons national surgical quality improvement program (ACS-NSQIP) registry. Patient Saf Surg. (2024) 18(1):29. 10.1186/s13037-024-00409-9 39354640 PMC11443812

[22] Peters NCJ Hijkoop A Lechner RL Eggink AJ Van Rosmalen J Tibboel D. The validity of the viscero-abdominal disproportion ratio for type of surgical closure in all fetuses with an omphalocele. Prenat Diagn. (2019) 39(12):1070–9. 10.1002/pd.5546 31410858 PMC6899735

[23] Peters NCJ Hooft ME Ursem NTC Eggink AJ Wijnen RMH Tibboel D. The relation between viscero-abdominal disproportion and type of omphalocele closure. Eur J Obstet Gynecol Reprod Biol. (2014) 181:294–9. 10.1016/j.ejogrb.2014.08.009 25201609

[24] Kleinrouweler CE Kuijper CF Van Zalen-Sprock MM Mathijssen IB Bilardo CM Pajkrt E. Characteristics and outcome and the omphalocele circumference/abdominal circumference ratio in prenatally diagnosed fetal omphalocele. Fetal Diagn Ther. (2011) 30(1):60–9. 10.1159/000323326 21325785

[25] Danzer E Victoria T Bebbington MW Siegle J Rintoul NE Johnson MP. Fetal MRI-calculated total lung volumes in the prediction of short-term outcome in giant omphalocele: preliminary findings. Fetal Diagn Ther. (2012) 31(4):248–53. 10.1159/000334284 22572017

[26] Dadoun SE Shanahan MA Parobek CM Burnett BA King A Ketwaroo P. Prenatal prognosis of omphalocele using magnetic resonance imaging measurement of fetal lung volumes. Am J Obstet Gynecol MFM. (2024) 6(10):101457. 10.1016/j.ajogmf.2024.101457 39098636

[27] Manohar K Mesfin FM Belchos J Brown BP Colgate C Timsina L. Beyond the womb: prenatal MRI’s prognostic abilities for morbidity and mortality in neonates with omphaloceles. J Perinatol. (2025) 45:1700–6. 10.1038/s41372-025-02321-1 40379902 PMC12716989

[28] Biard J-M Wilson RD Johnson MP Hedrick HL Schwarz U Flake AW. Prenatally diagnosed giant omphaloceles: short- and long-term outcomes. Prenat Diagn. (2004) 24(6):434–9. 10.1002/pd.894 15229842

[29] Vachharajani AJ Rao R Keswani S Mathur AM. Outcomes of exomphalos: an institutional experience. Pediatr Surg Int. (2009) 25(2):139–44. 10.1007/s00383-008-2301-y 19066916

[30] Teillet B Boukhris MR Sfeir R Mur S Cailliau E Sharma D. Systemic inflammation is associated with pulmonary hypertension in isolated giant omphalocele: a population-based study. Healthcare (Basel). (2022) 10(10):1998. 10.3390/healthcare10101998 36292445 PMC9601560

[31] Llop J Badía MB Comas D Tubau M Jódar R. Colonization and bacteremia risk factors in parenteral nutrition catheterization. Clin Nutr. (2001) 20(6):527–34. 10.1054/clnu.2001.0488 11884001

[32] Nakayama DK Mutich R Motoyama EK. Pulmonary dysfunction after primary closure of an abdominal wall defect and its improvement with bronchodilators. Pediatr Pulmonol. (1992) 12(3):174–80. 10.1002/ppul.1950120309 1386420

[33] Dimitriou G Greenough A Kavvadia V Davenport M Nicolaides KH Moxham J. Diaphragmatic function in infants with surgically corrected anomalies. Pediatr Res. (2003) 54(4):502–8. 10.1203/01.PDR.0000081299.22005.F0 12815114

[34] Reighard C Junaid S Jackson WM Arif A Waddington H Whitehouse AJO. Anesthetic exposure during childhood and neurodevelopmental outcomes: a systematic review and meta-analysis. JAMA Netw Open. (2022) 5(6):e2217427. 10.1001/jamanetworkopen.2022.17427 35708687 PMC9204549

